# Function Analysis of a Maize Endo-1,4-β-xylanase Gene *ZmHSL* in Response to High-Temperature Stress

**DOI:** 10.3390/ijms25168834

**Published:** 2024-08-14

**Authors:** Shengyan Pang, Hongyan Zheng, Jiankui Zhang, Xiaotian Ren, Xuefeng Zong, Junjie Zou, Lei Wang

**Affiliations:** 1 Functional Genome Research Center, Biotechnology Research Institute, Chinese Academy of Agricultural Sciences, Beijing 100081, China; 18786777149@163.com (S.P.); zhenghongyan@caas.cn (H.Z.); rxt1600@163.com (X.R.); 2 College of Agronomy and Biotechnology, Southwest University, Chongqing 400715, China; jkzhang@swu.edu.cn (J.Z.); zxfeng@swu.edu.cn (X.Z.); 3 College of Bioscience and Resources Environment, Beijing University of Agriculture, Beijing 102206, China

**Keywords:** BSA-seq, cell wall, endo-β-1,4-xylanase, heat stress, maize, RNA-seq, water transport

## Abstract

Rising temperature is a major threat to the normal growth and development of maize, resulting in low yield production and quality. The mechanism of maize in response to heat stress remains uncertain. In this study, a maize mutant *Zmhsl-1* (*heat sensitive leaves*) with wilting and curling leaves under high temperatures was identified from maize Zheng 58 (Z58) mutant lines generated by ethyl methanesulfonate (EMS) mutagenesis. The *Zmhsl-1* plants were more sensitive to increased temperature than Z58 in the field during growth season. The *Zmhsl-1* plants had lower plant height, lower yield, and lower content of photosynthetic pigments. A bulked segregant analysis coupled with whole-genome sequencing (BSA-seq) enabled the identification of the corresponding gene, named *ZmHSL*, which encodes an endo-β-1,4-xylanase from the GH10 family. The loss-of-function of *ZmHSL* resulted in reduced lignin content in *Zmhsl-1* plants, leading to defects in water transport and more severe leaf wilting with the increase in temperature. RNA-seq analysis revealed that the differentially expressed genes identified between Z58 and *Zmhsl-1* plants are mainly related to heat stress-responsive genes and unfolded protein response genes. All these data indicated that *ZmHSL* plays a key role in lignin synthesis, and its defective mutation causes changes in the cell wall structure and gene expression patterns, which impedes water transport and confers higher sensitivity to high-temperature stress.

## 1. Introduction

Maize (*Zea mays* L.) is a high-yielding temperate crop providing the world with nutritious food, feed, and industrial feedstocks. Sustainable production of maize is crucial for food security. As global warming continues to increase, high temperature has become a serious threat to maize growth and productivity [1]. High temperature usually causes numerous deleterious effects on maize, such as inhibition of photosynthesis, fertility reduction, oxidative damage, and shorter vegetative and reproductive phases, cumulatively resulting in a significant yield loss [1,2]. Therefore, the genetic improvement of high-temperature resistance in maize is becoming increasingly desirable.

Plants have evolved complex responses to heat stress to minimize damage via activating multiple signal transduction pathways [2]. Genetic, physiological, molecular, and biochemical studies have identified some vital cellular components and processes involved in heat-responsive growth and the acquisition of thermotolerance in plants [3]. Heat stress induces a cytoplasmic heat stress response (HSR), causes the endoplasmic reticulum (ER)-localized unfolded protein response (UPR), and also activates hormone responses and alternative RNA splicing, all of which may contribute to heat stress tolerance [4]. Maize HEAT SHOCK FACTOR A2 (HSFA2) and HEAT SHOCK BINDING PROTEIN 2 (HSBP2) physically interact with each other and antagonistically modulate raffinose biosynthesis and heat tolerance [5]. Maize heat shock protein 101 (HSP101) mediates thermotolerance during microsporogenesis, playing an important role in microspore adaption to high temperatures in maize [6]. The maize transcription factor ZmNAC074 acts as a positive regulator that activates the expression of ROS-scavenging genes and HSR- and UPR-associated genes to enhance plant heat stress tolerance [7]. *ZmHUG1* (*HEAT UPREGULATED GENE1*) encodes an ER-localized chaperone and is induced by heat stress. The *zmhug1* mutant displays hypersensitivity to heat stress and more severe ER stress [2].

Plant cell walls are highly dynamic structures and function in providing mechanical support for plant cells during growth, development, and adaptation to environmental stresses [7]. Plant cell wall components and structure change in response to environmental stress, altering their mechanical properties and resilience to environmental cues. For example, the chemical profile of coffee cell wall polymers and structural cell anatomy in coffee leaves change under heat stress [8]. In tomato leaves, β-glucosidase participates in heat stress by degrading cellulose [9]. The xyloglucan transglucosidase XET/XTH family is involved in plant cell wall extensibility, and most genes in this family are upregulated by stress induction, thereby participating in plant response to abiotic stress [10,11]. Transgenic overexpressing pepper *XTH3* in tomatoes ameliorated salt tolerance [12]. Arabidopsis (*Arabidopsis thaliana*) cellulose synthase gene *AtCesA8* acts as a key enzyme in cellulose biosynthesis; the loss-of-function mutant, which features xylem collapse in roots and stems, is more tolerant to drought stress, as well as to NaCl, mannitol, and other osmotic stresses [13]. Expansins (EXPs) in the cell walls play an important role in plant adaptation to abiotic stresses. For example, the expression levels of numerous *EXP* genes in maize, including *ZmEXPA1*, *ZmEXPA3*, *ZmEXPA5*, *ZmEXPB1*, and *ZmEXPB2,* were upregulated by salt treatment, concurrent with the hydrolysis and reconnection of xylose molecules in the cell wall [14]. Likewise, under heat stress, the overexpression of Bluegrass *PpEXP1* in tobacco alleviated the damage to the cell structure and improved heat resistance compared with wild-type plants [15]. Altogether, these studies have demonstrated that cell wall changes caused by defective cell wall-related proteins or enzymes are involved in plants responding to environmental stresses.

Xylan is the main chemical component of hemicellulose, an important part of plant cell walls. Endo-β-1,4-xylanases are the major members of the plant GH family 10 (GH10) and play important roles in the enzymatic hydrolysis of xylan, which preferentially acts on long-chain oligosaccharides and xylan from different sites, cleaves the xylosidic bonds inside the main chain of β-1,4-xylan, and degrades xylan into xylobiose and xylo-oligosaccharides that are longer than xylobiose. The endo-β-1,4-xylanase of barley may play an important role in programmed cell death (PCD) in the aleurone layer [16]. Arabidopsis xylanase gene *AtXyn1* is mainly expressed in vascular cells, and its overexpression in stems resulted in a two-fold increase in enzyme activity compared to the wild type, suggestive of its potential role in secondary cell wall metabolism of vascular cells [17]. In papaya, xylanase may be associated with fruit softening during ripening [18]. In rice, a loss-of-function mutant of xylanase *OsXyn1* exhibited abnormality in the intermediate layer of the cell wall, resulting in impaired water transport [19].

High temperature can cause a series of changes in plant cell walls at morphological, physiological, biochemical, and molecular levels, which results in stunted growth, reduced yield, and even death in severe cases [20,21,22]. However, the information on the key genes regulating cell wall changes under high-temperature stress in maize is still limited [10,23,24]. In this study, one *ZmHSL* encoding an endo-β-1,4-xylanase was identified and played an important role in cell wall metabolism and water transport in response to increased ambient temperature. The leaves of *Zmhsl-1* plants became wilting and curling with the increase in ambient temperature. The *Zmhsl-1* plants contained reduced lignin content, leading to impaired water transport. Additionally, comparative transcriptomic analysis enabled the identification of heat resistance-related genes, providing important clues for molecular breeding of high-temperature resistance in maize.

## 2. Results

### 2.1. Phenotypic Analysis of a Maize Heat-Sensitive Mutant Zmhsl-1

**Figure 1:**
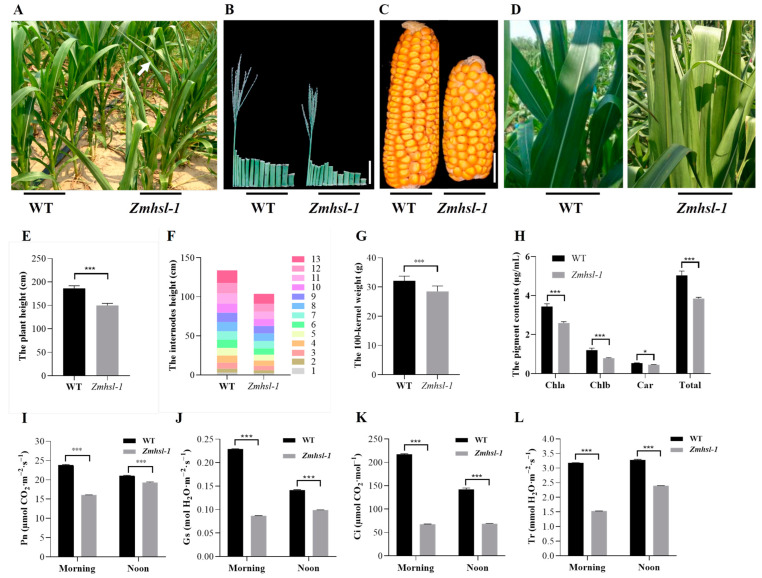
Phenotypic comparison between wild-type and *Zmhsl-1* plants. (**A**) Phenotypes of wild-type (WT) and *Zmhsl-1* plants at late whorl stage. The arrow indicates the curling leaf. (**B**) Internodes and tassel of wild-type and *Zmhsl-1* plants at tasselling stage, Bar = 10 cm. (**C**) Ears of wild-type and *Zmhsl-1* plants at the mature stage, bar = 3 cm. (**D**) Comparison of leaf colour between wild-type and *Zmhsl-1* plants. (**E**) Comparison of plant height between wild-type and *Zmhsl-1* plants, *n* = 18. (**F**) Comparison of internode length between wild-type and *Zmhsl-1* plants, *n* = 10. (**G**) Comparison of 100-grain weight between wild-type and *Zmhsl-1*, *n* = 10. (**H**) Determination of pigment content of wild-type and *Zmhsl-1* leaves. (**I**) Determination of net photosynthetic rate (Pn) between wild-type and *Zmhsl-1* plants. (**J**) Determination of stomatal conductance (Gs) between wild-type and *Zmhsl-1* plants. (**K**) Determination of intercellular carbon dioxide concentration (Ci) between wild-type and *Zmhsl-1* plants. (**L**) Measurement of respiration rate (Tr) between wild-type and *Zmhsl-1* plants. *n* represents the number of samples; *t*-test (* *p* < 0.05, *** *p* < 0.001).

*Zmhsl-1* is a high temperature-sensitive mutant obtained from the mutant library of maize inbred line Zheng58 (Z58) generated by EMS mutagenesis. Under normal field growth conditions, the leaf curling in *Zmhsl-1* was barely noticeable in the morning and later afternoon during their vegetative growth period, in stark contrast to the obvious curling observed when the ambient temperature was higher than 30 °C at noon time during the day (Figure 1A and Figure S1). When grown in a controlled growth chamber, there was no difference between *Zmhsl-1* and wild-type plants at the seedling stage. After treatment at 42 °C for 6 h, *Zmhsl-1* plants showed a sensitive type compared to wild-type plants, with leaf wilting and curling. In contrast, the leaves of wild-type plants remained erect even after treatment for 24 h (Figure S2). All these data indicated that *Zmhsl-1* is more sensitive to high-temperature stress than wild-type plants. 

When grown in the field at the silking stage, the *Zmhsl-1* plants exhibited significantly reduced plant height, which was attributable to the reduced node length despite the number of nodes that remained unaltered relative to Z58 (Figure 1B,E,F). Likewise, *Zmhsl-1* plants were also featured, with significantly shorter ear length, lower one hundred-kernel weight, smaller ear diameter, lower row number per ear, and lower kernel number per row in comparison with Z58 at the harvest stage (Figure 1C,G and Figure S3A–D). Moreover, the leaf colour of *Zmhsl-1* was lighter than Z58, which was corroborated by the reductions in the contents of chlorophyll a, chlorophyll b, carotenoids, and total pigments in *Zmhsl-1* leaves (Figure 1D,H). Conceivably, photosynthesis (Pn), stomatal conductance (Gs), internal CO_2_ concentration (Ci), and transpiration rate (Tr) in *Zmhsl-1* were all significantly lower than in wild-type plants (Figure 1I–L). 

### 2.2. Fine Mapping and Identification of the Candidate Gene of Zmhsl-1

All the F_1_ plants obtained by crossing *Zmhsl-1* with Z58 showed the wild-type phenotype during their growth period in the field. When encountering high-temperature weather (higher than 30 °C) in the summer, the square test results show that the ratio of erect leaf plants to curling leaf plants in the F_2_ population followed the anticipated 3:1 separation ratio, indicating that the curling leaf phenotype of *Zmhsl-1* was ruled by a single recessive gene (χ^2^ < 3.84) (Table 1). The square test of the segregation ratio of the BC1F1 population derived from the backcrossing of F_1_ by *Zmhsl-1* indicated that the ratio of normal leaf plants to curling leaf plants conformed to the 1:1 segregation rule (χ^2^ < 3.84) (Table 1), corroborating the F_2_ segregation results that *Zmhsl-1* is a single-gene recessive mutant.

**Figure 2:**
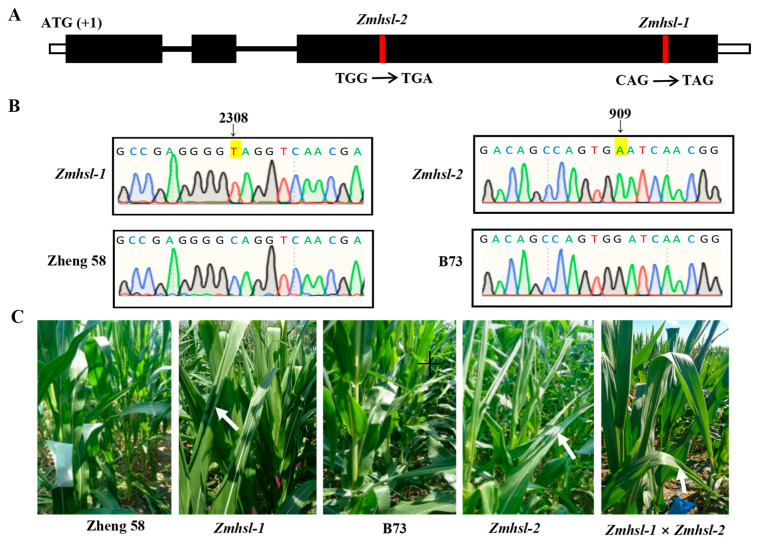
*ZmHSL* mutation site and allele test. (**A**) Schematic diagram of *ZmHSL* mutation site. (**B**) *Zmhsl-1* and *Zmhsl-2* sequencing peak map. (**C**) The phenotype of F_1_ plants derived from the cross of *Zmhsl-1* with *Zmhsl-2* at noon in the field with a temperature higher than 30 °C. The arrows indicate curling leaves.

To identify the mutant gene of *Zmhsl-1*, a bulked segregant analysis coupled with the whole-genome sequencing (BSA-Seq) method was conducted [25]. The fifty-five *Zmhsl-1* plants with curling leaves and thirty-five plants with erect normal leaves in the F_2_ generation (Zheng58 × *Zmhsl-1*) were separated and used for sequencing. The results show that the causative gene of *Zmhsl-1* is located on chromosome 5. There are two candidate genes, *Zm00001d013540* and *Zm00001d013561*, in the selected mapping interval (Table 2). In *Zm00001d013561*, a single nucleotide substitution C2038T represents a C to T conversion at the 2038 bp from the start codon (first ATG) of the gene coding sequence, resulting in premature termination of protein translation, indicating that *Zm00001d013561* may be the target gene of *Zmhsl1-1* (Figure 2A). To confirm this, its corresponding mutant line, *Zmhsl-2* (stop_gained), ordered from the maize B73 EMS mutant library (http://maizeems.qlnu.edu.cn/ accessed on 25 October 2018), exhibited a similar phenotype resembling *Zmhsl-1* (Figure 2B). The F_1_ plants obtained by crossing *Zmhsl-2* with *Zmhsl-1* showed a curling leaf phenotype under the high-temperature condition, similar to *Zmhsl-1* and *Zmhsl-2* single mutants (Figure 2C), indicating that *Zm00001d013561* is the causative gene in *Zmhsl-1*.

### 2.3. ZmHSL Encodes Endo-1,4-β-xylanase

**Figure 3:**
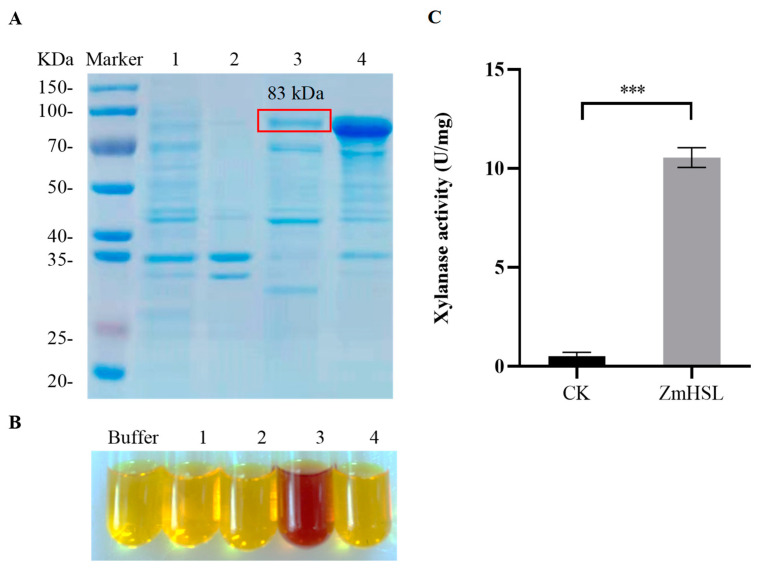
Determination of ZmHSL xylanase activity. (**A**) SDS-PAGE electrophoresis picture of ZmHSL expressed protein. (**B**) Xylanase activity of ZmHSL protein was determined by 3,5-dinitrosalicylic acid. (**C**) Xylanase activity of ZmHSL and control; the error bars represent ± SE (*n* = 3), *t*-test (***, *p* < 0.001). The numbers “1, 2, 3, and 4” in (**A**,**B**) represent pET-28a(+) unloaded supernatant, pET-28a(+) unloaded precipitate, pET-28a(+)-*ZmHSL* supernatant, and pET-28a(+)-*ZmHSL* precipitate.

A database query revealed that *ZmHSL* belongs to the GH10 family, the main member of which is annotated as endo-1,4-β-xylanase [26,27,28]. The expression of *ZmHSL* with a prokaryotic expression vector pET-28a(+)-*ZmHSL* in *E. coli* resulted in the induction of its active protein product. SDS-PAGE gel analysis showed the appearance of a novel band of approximately 83 kDa in both the supernatant and the pellet fractions after induction (Figure 3A), indicating that the ZmHSL is successfully expressed in the prokaryotic expression host BL21. There was more abundant gene product in the pellet fraction than in the supernatant. As shown in Figure 3B,C, the product in the supernatant was enzymatically active, with 10.5 U/mg endo-1,4-β xylanase activity, which was significantly higher than that of the empty vector control. In contrast, the fraction in the precipitate fraction had no discernible enzyme activity (Figure 3B,C).

### 2.4. ZmHSL Functions in Regulating Cell Wall Content and Then Water Transport

**Figure 4:**
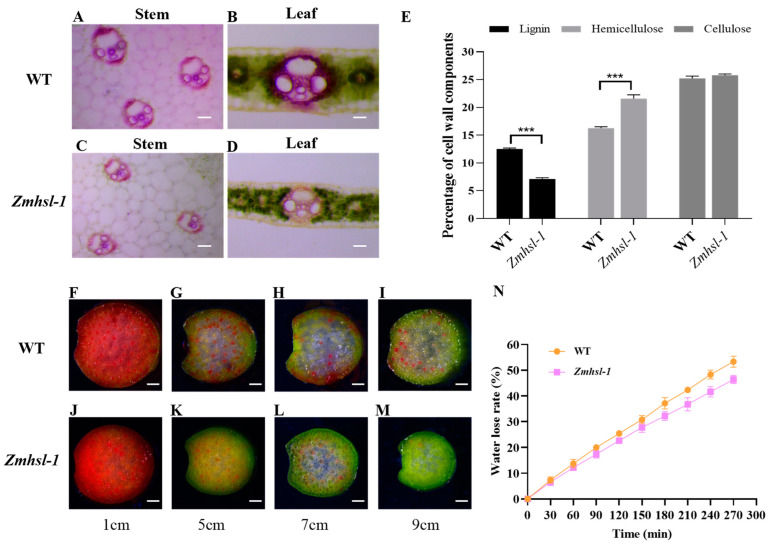
Comparison of cell wall components and water transport between wild-type and *Zmhsl-1* plants. (**A**–**D**) Lignin staining of cross-sections of stems and leaves from wild-type and *Zmhsl-1* plants. (**A**,**C**) Bar = 0.1 mm, (**B**,**D**) Bar = 0.2 mm. (**E**) Determination of cell wall components in wild-type and *Zmhsl-1* plants. (**F**–**M**) Cross-section staining observations at 1 cm, 5 cm, 7 cm, and 9 cm above the stained end of a stem in wild-type and *Zmhsl-1* plants. (**N**) The detached leaf water loss rates of wild-type and *Zmhsl-1* plants at indicated time. *t*-test (*** *p* < 0.001).

*ZmHSL* encodes an endo-1,4-β-xylanase and is expressed in various tissues, with the highest expression level in stems at the tasselling stage (Figure S4). Lignin staining of the leaf and stem cross sections with phloroglucinol reagent showed that the staining of the sclerenchyma cells is conspicuously lighter in *Zmhsl-1* (Figure 4C,D) than in wild-type plants (Figure 4A,B). Quantitative analysis of cell wall components in leaves revealed that *Zmhsl-1* plants have significantly lower lignin content and significantly higher hemicellulose content compared with wild-type plants, while the cellulose content remained unaltered (Figure 4E). Water is transported from roots to leaves through the plant xylem, and damage to the xylem may hinder water transport [29]. To evaluate the water transport capacity of *Zmhsl-1* plants, red ink staining was performed by using the stems of wild-type and *Zmhsl-1* plants grown for six weeks. Analysis of the position of the red ink in the stem at the indicated distance showed that there is a substantial defect in stem water transport in *Zmhsl-1* plants compared to the wild type (Figure 4F–M). Further, an experiment on water loss of detached leaves of six-week-old wild-type and *Zmhsl-1* plants revealed that the rate of water loss of leaves of *Zmhsl-1* plants is slower than that of wild-type plants (Figure 4N). Taken together, these results suggest that the loss of function of *ZmHSL* results in the alteration of cell wall components in *Zmhsl-1* plants, which then leads to impaired water transport.

### 2.5. Differently Expressed Genes between Wild Type and Zmhsl-1 by Transcriptome Analysis

#### 2.5.1. Functional Enrichment Analysis of Differentially Expressed Genes

**Figure 5:**
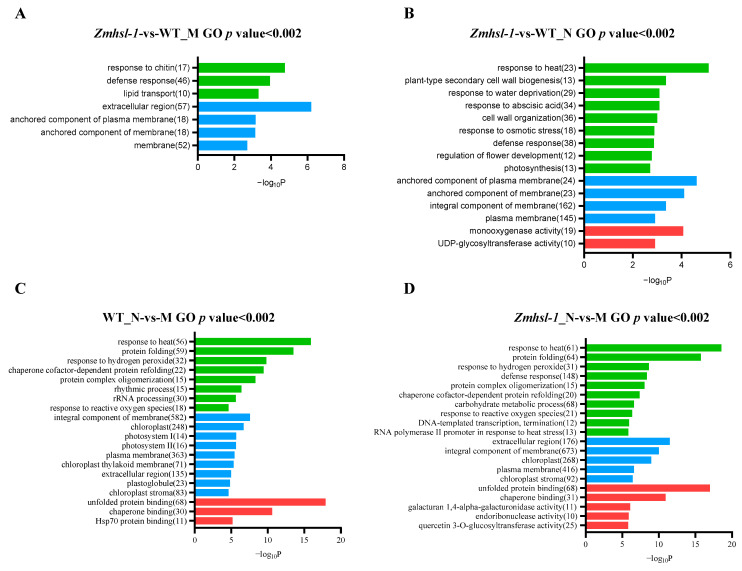
GO enrichment analysis of wild-type and *Zmhsl-1* plants. (**A**) GO enrichment analysis of DEGs between wild type and *Zmhsl-1* at 24 °C (in the morning). (**B**) GO enrichment analysis of DEGs between wild type and *Zmhsl-1* at 34 °C (at noon). (**C**) GO enrichment analysis of DEGs in wild-type plants between morning and noon. (**D**) GO enrichment analysis of DEGs in *Zmhsl-1* between morning and noon. The M in the picture refers to “morning” with an ambient temperature of about 24 °C, and N refers to “noon” with an ambient temperature of about 34 °C.

With the increase in ambient air temperature, the leaves of *Zmhsl-1* plants became curling compared to Z58. To analyse the difference in gene expression levels between *Zmhsl-1* and Z58 plants, leaves above the ear of *Zmhsl-1* and Z58 plants were collected in the morning (about 24 °C, represented by M) and at noon (about 34 °C, represented by N) at the tasselling stage for RNA-seq. A total of 12 samples were collected, and three biological replicates were set for each. After quality inspection, adapters, low-quality sequences, and ambiguous readings were removed. A total of 91.26 G of CleanData was obtained, with effective data volumes ranging from 7.24 to 8.17 G for each sample and Q30 bases ranging from 95.26 to 96.06%. The average GC content was 54.95%. By comparing reads to the reference genome, the genome alignment of each sample was obtained, with alignment rates ranging from 90.60% to 93.34% (Table S1). The *p* value < 0.05 and the fold change (Fold Change) satisfying |log_2_FoldChange| > 1 were used as the screening criteria for differentially expressed genes (DEGs). There were a total of 1313 DEGs between *Zmhls-1* and Z58 at 24 °C in the morning, of which 932 DEGs were upregulated and 381 DEGs were downregulated (Figure S5). The top three main enriched GO terms were “extracellular region” (GO: 0005576), “defence response” (GO: 0006952), and “response to chitin” (GO: 0010200) (Figure 5A). Moreover, there were a total of 2072 DEGs between *Zmhls-1* and Z58 under 34 °C (at noon), of which 1259 DEGs were upregulated and 813 DEGs were downregulated (Figure S5). The first three main enriched GO terms were “the response to heat stress” (GO: 0009408), “anchoring components of the plasma membrane” (GO: 0046658), and “monooxygenase activity” (GO: 0004497) (Figure 5B). The number of DEGs between Z58 and *Zmhsl-1* at noon under the high-temperature condition was more than those in the morning under normal ambient temperatures, and the main enrichment process was significantly different, indicating that *ZmHSL* was involved in maize response to high-temperature stress.

Compared with an ambient temperature of about 24 °C in the morning, under the high temperature of about 34 °C at noon, a total of 6382 genes were differentially expressed in wild-type Z58, of which 3098 DEGs were upregulated and 3284 DEGs were downregulated (Figure S5). A total of 7006 genes were differentially expressed in *Zmhsl-1*, of which 3250 DEGs were upregulated and 3756 DEGs were downregulated (Figure S5). GO functional enrichment analysis showed that the top three GO terms related to molecular function were the same in the wild type and *Zmhsl-1* when induced by mid-day high temperatures. The top three GO terms related to cell composition in wild-type Z58 were “membrane component” (GO: 0016021), “chloroplast” (GO: 0009507), and “photosystem I” (GO: 0009522). The top three GO terms related to cell composition in *Zmhsl-1* were “extracellular region” (GO: 0005576), “membrane component” (GO: 0016021), and “chloroplast” (GO: 0009507). The top three GO terms related to biological processes in the wild type were “unfolded protein binding” (GO: 0051082), “chaperone protein binding” (GO: 0051087), and “HSP70 protein binding” (GO: 0030544). The top three GO terms related to the biological process in *Zmhsl-1* were “unfolded protein binding” (GO: 0051082), “chaperone binding” (GO: 0051087), and “α-galactosidase activity” (GO: 0047911) (Figure 5C,D).

#### 2.5.2. Expression Analysis of HSF-, HSP-, and UPR-Related Genes in Wild-Type and *Zmhsl-1* Plants under High-Temperature Stress

**Figure 6:**
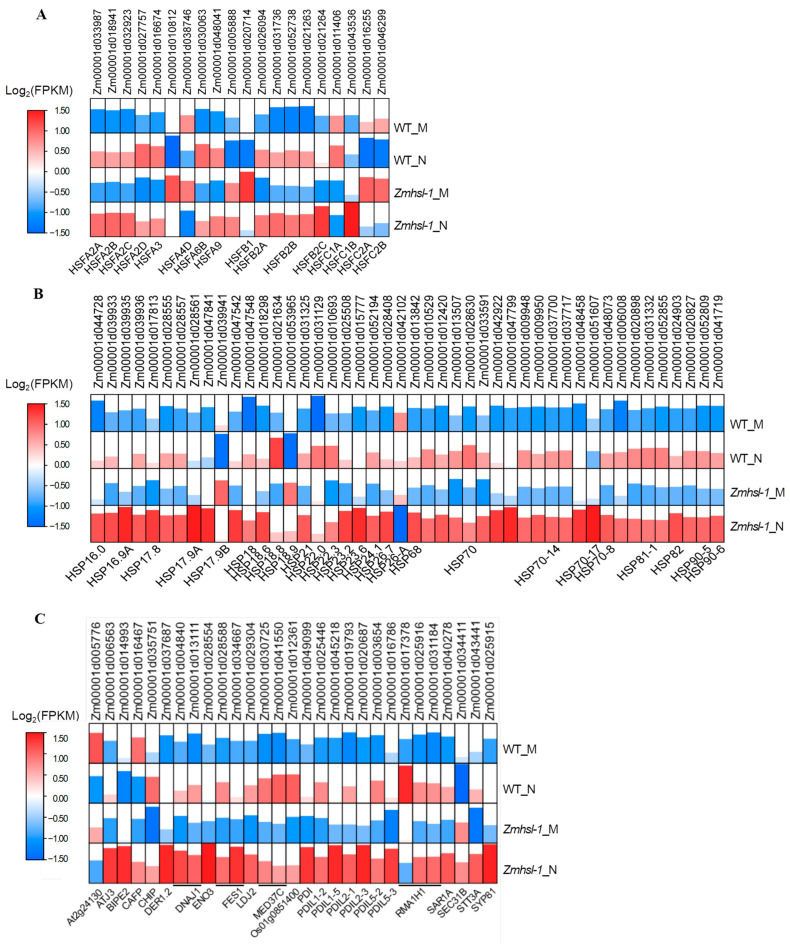
Expression patterns of HSR- and UPR-related genes in different maize genotypes. (**A**) Expression pattern of DEGs associated with the heat shock transcription factors (HSFs). (**B**) Expression pattern of DEGs associated with the heat shock proteins (HSPs). (**C**) Expression pattern of DEGs associated with unfolded protein response (UPR).

Heat shock transcription factors (HSFs) and their regulated heat shock proteins (HSPs) play important roles in plants responding to high-temperature stress and withstanding the damages caused by high temperature. Using mining and analysis of HSF- and HSP-related genes in the transcriptome data, a total of 20 *HSF*- and 46 *HSP*-related genes were found to be DEGs, most of which were upregulated by high temperature induction, and the expression levels in *Zmhsl-1* were higher than those in wild-type plants (Figure 6A,B).

In response to high-temperature stress, plants also activate the unfolded protein response (UPR) in the ER to alleviate cellular damages inflicted by high temperatures [30]. This is reflected by the enrichment of unfolded protein binding (GO: 0051082) in the DEGs under high temperatures in both wild-type and *Zmhsl-1* plants (Figure 5C,D). A total of 29 typical *UPR* genes were identified, most of which were upregulated by high temperature induction, and the expression level in *Zmhsl-1* was higher than that in wild-type plants (Figure 6C).

## 3. Discussion

### 3.1. Alterations in Cell Wall Structure and Composition Lead to Defects in Water Transport

The cell wall is mainly composed of cellulose, hemicellulose, lignin, pectin, and other substances. Proper cell wall structure provides mechanical support and load-bearing capacity, enabling the transport of water and nutrients in plants over long distances [23]. Arabidopsis *CESA* genes, including *AtCesA4*, *AtCesA7*, and *AtCesA8*, have been well characterized, their loss-of-function mutation having resulted in xylem collapse, water transport blockage, and leaf wilting [13,31,32]. Maize *nut1-m1*, a leaf wilting mutant caused by the insertion of an Ac transposon into the coding region of the NAC transcription factor, has a thinner xylem cell wall and reduced water transport capacity. It has been speculated that NUT1 may play a role in regulating the thickness and strength of the xylem cell walls [33]. Likewise, mutagenesis of *OsXyn1* in rice resulted in the formation of an aberrant cell wall intermediate layer and blockage of water transport [19]. In this study, we cloned and identified a heat-sensitive gene, *ZmHSL*, that encodes an endo-β-1,4-xylanase capable of hydrolysing xylan, as reported before [34]. Its loss-of-function mutant, *Zmhsl-1*, is endowed with alterations in cell wall structure and composition (Figure 4A–E). These structural and compositional changes have contributed to weakened xylem structural strength, hindered xylem water transport, and reduced rate of leaf water loss (Figure 4F–N). Under heat stress, the *Zmhsl-1* plants exhibit the leaf curling and wilting phenotype due to inadequate water transport to cope with the increased transpiration rate.

### 3.2. ZmHSL Is Essential for Maize Normal Growth and Development

In an Arabidopsis *Brassinosteroid* mutant, *CESA* expression was reduced, along with reduced cellulose content and altered cell wall structure, which renders it insufficient to support normal cell development, thus resulting in stunted plant growth and development [35]. Likewise, the loss-of-function mutation in the lignin synthesis-related genes, including cinnamic acid-4-hydroxylase (*C4H*), cinnamoyl-CoA reductase (*CCR*), and phenylalanine ammonialyase (*PAL*), resulted in lignin reduction and dwarfed plant formation [36,37,38]. In rice *xyn1* mutant, an abnormality in the middle layer of the cell wall was observed, which was associated with obstructions in water transport and significantly reduced plant height [19]. In the present study, the *Zmhsl-1* mutant showed shorter internodes and reduced plant height compared to wild-type plants (Figure 1). Further, the lignin content of *Zmhsl-1* plants was significantly reduced (Figure 4A–E). The loss of *ZmHSL* function leads to a significant decrease in lignin content and weakening of the structural strength of the xylem, resulting in hindered water transport, which is exacerbated by high-temperature stress. When *Zmhsl-1* plants were exposed to high-temperature stress, the photosynthetic pigments and photosynthetic parameters in leaves were significantly reduced, accompanied by fading of leaf colour. The leaf is the main plant organ carrying out photosynthetic carbon assimilation, which plays a crucial role in plant growth and development as an important source of dry matter accumulation that forms the basis for grain yield [39,40,41]. In this study, *Zmhsl-1* plants exhibited significantly reduced ear length, ear diameter, ear row number, grain number per row, and 100-grain weight (Figure 1 and Figure S3), likely due to impaired photosynthesis. In addition, *ZmHSL* was highly expressed in developing maize seeds at the grain filling and full maturity stages (Figure S4); therefore, it is conceivably speculated that *ZmHSL* may also directly affect grain development.

### 3.3. The Underlying Mechanism of Heat Sensitivity in ZmHSL

HSFs are the most important regulators for plant responses and resistance to high-temperature stress by activating *HSP* expression [42,43]. The HSFs reported to date include HSFAs, DREB, bZIP, and WRKY, among which HSFA1 and HSFA2 are the main transcriptional regulators in response to high-temperature stress and play key roles in plant transcriptional regulatory networks in response to high-temperature stress [44,45,46]. In Arabidopsis, the deletion mutant of *HSFA1* is characterized by a heat-sensitive phenotype and attenuated expression of heat-responsive genes, whereas the overexpression of *HSFA1* in tomatoes exhibited enhanced heat tolerance [47,48]. HSPs are among the proteins regulated by HSFs which are involved in regulating plant cell membrane homeostasis and heat stress-related metabolic processes in response to high-temperature stress [49,50]. Overexpression of *HSP90* in rice and soybean was shown to increase heat tolerance in these crops [51,52]. High-temperature stress can also cause ER stress and trigger UPR response, alleviating the damage to plants inflicted by heat stress by improving the protein folding capacity in the ER cavity and accelerating the degradation of misfolded proteins [30]. A recent study showed that maize ZmHSF20 acts as a negative regulator of heat stress; it can inhibit cellulose accumulation and repress the expression of cell wall-related genes, revealing the importance of the structure of cell wall change in response to heat [53]. In this study, the expression of *HSF*-, *HSP*-, and *UPR*-related genes in *Zmhsl-1* was substantially higher than that in wild-type plants under high-temperature conditions (Figure 6). The reason for this result may be that the loss of *ZmHSL* function leads to changes in the xylem structural strength of the mutant, resulting in water transport blockage and thereby leading to increased leaf temperature in *Zmhsl-1*. With increasing growth temperature, transpiration is enhanced, which renders greater damage to *Zmhsl-1* than to the wild-type plants. Apart from the reported *HSFA1s* and *HSFA2s,* which play a major regulatory role in heat tolerance, *HSFB2A*, *HSFB2B*, *HSFB2C,* and *HSFC1B* genes were also significantly upregulated by high-temperature stress (Figure 6A), suggesting their potential functional roles in response to high-temperature stress in maize leaves. Additionally, the DEGs found between wild-type and *Zmhsl-1* plants could result from other outdoor factors (e.g., light conditions) or their interactions, increasing the complexity of the molecular mechanism of *ZmHSL-1* in response to heat stress.

## 4. Materials and Methods

### 4.1. Plant Materials

The *Zmhsl-1* mutant was isolated from Z58 mutant lines generated by EMS. The mutant *Zmhsl-2* in B73 background was ordered from the MEMD library (http://elabcaas.cn/memd/public/index.html#/pages/search/geneid accessed on 25 October 2018) [54].

### 4.2. Measurements of Agronomic Traits

To measure the agronomic traits of the Z58 wild-type, *Zmhsl-1* and *Zmhsl-2*, the plants were grown in the field at the Wanzhuang Agricultural Research Station of the Chinese Academy of Agricultural Sciences (CAAS), Langfang, Hebei Province, China, as well as at Nanfan experimental base in Hainan Province, China. In Langfang, the seeds of different materials were sown in early May, and the min and max air temperatures in summer were 22 °C and 36 °C, respectively. Agronomic traits, including leaf phenotype, plant height, ear length, ear diameter, number of rows per ear, number of grains per row, and 100-grain weight, were measured. The significance of variation among different samples was analysed by Student’s *t*-test.

To observe the phenotype of *Zmhsl-1* under high temperatures in a controlled condition, the wild-type and *Zmhsl-1* plants were grown in a controlled growth chamber with 28 °C/25 °C day/night temperature cycles and 16 h/8 h light/dark illumination cycles. At the three-leaf stage, the wild-type and *Zmhsl-1* plants were treated at 42 °C, and the plants were photographed at different time points.

### 4.3. Determination of Photosynthetic Pigments and Photosynthetic Parameters

At the tasselling stage, the penultimate fifth leaf in the wild-type and *Zmhsl-1* mutant plants was harvested and washed, and then the main veins were removed. The chlorophyll was extracted with 95% ethanol, and its absorbance was measured at wavelength 470, 649, 652, and 665 nm absorbance value by using a UV-8000S spectrophotometer (Shanghai Yuanxi Instrument Co., Ltd., Shanghai, China). The contents of various photosynthetic pigments, including chlorophyll a (Chla), chlorophyll b (Chlb), and carotenoid (Car), were calculated by referring to the method described by Wellburn [55]. We analysed the content of chlorophyll a, chlorophyll b, carotenoids, and total chlorophyll in different materials based on wavelength values and related calculation formulas, as follows: Ca = 13.95A_665_ − 6.88A_649_; Cb = 24.96A_649_ − 7.32A_665_; Cc = (1000A_470_ − 2.05Ca − 114.8Cb)/245; chlorophyll content (μg/g) = (concentration of each chlorophyll x volume of extraction solution)/weight of leaf fragments; total chlorophyll content (mg/g) = (A652 × volume of extraction solution)/(34.5 × weight of leaf fragments). Among these, Ca represents the concentration of chlorophyll a (μg/mL), Cb represents the concentration of chlorophyll b (μg/mL), and Cc represents the concentration of chlorophyll c.

In the morning (about 24 °C) and at noon (about 34 °C) on a sunny day, the LI-6400 portable photosynthesis apparatus (LI-COR Inc., Lincoln, NE, USA) equipped with 6400-02B red and blue light sources (LI-COR Inc., Lincoln, NE, USA) was used to measure the photosynthetic parameters of Z58 and *Zmhsl-1* in the penultimate fifth leaf at the grain filling stage. The measured photosynthetic parameters included the net photosynthetic rate (Pn), stomatal conductance (Gs), intercellular CO_2_ concentration (Ci), and transpiration rate (Tr). 

### 4.4. Genetic Analysis of Mutants

Crosses were made by using Z58 as a male parent and *Zmhsl-1* as a female parent, and the F_2_ population was obtained by self-pollination. F_1_ plants were also backcrossed with *Zmhsl-1* to obtain BC_1_F_1_. The F_2_ and BC_1_F_1_ seeds were sown in the field. The number of plants with normal leaves or curling leaves was counted at the V6 stage in the field when the ambient air temperature was higher than 30 °C.

### 4.5. BSA-Seq Analysis

DNA samples were prepared from the leaves derived from 55 individual plants similar to the *Zmhsl-1* phenotype in the aforementioned F_2_ population and 35 individual plants similar to the Z58 phenotype, which were mixed prior to being subjected to BSA-seq in OE Biotech Co., Ltd. (Shanghai, China) by following standard protocols. Sequence alignment, gene annotation, SNP analysis, candidate gene identification, and localization analysis were conducted as previously described [56,57]. The libraries were constructed with the TruSeq Nano DNA LT Sample Preparation Kit (Illumina, San Diego, CA, USA). The raw reads were subjected to a quality check and then filtered by fastp tool. Clean reads were aligned to the reference genome using the Burrows–Wheeler Aligner (BWA, Version 0.7.12) with the default options. The mapped reads were sorted and indexed by using SAMtools (Version: 1.4). GATK (Version 4.1.0.0) was used to call out all the variants, including SNPs and InDels. Then, SnpEff software (version 4.1g) was applied to annotate all the variants. 

### 4.6. Xylanase Activity Assay

To analyse whether ZmHSL has xylanase, the coding region of *ZmHSL* was cloned and inserted into expression vector pET-28a, which was expressed in *E. coli* strain BL21 (DE3). The recombinant protein was obtained after induction with 0.1 mM isopropyl-β-D-thiogalactoside (IPTG) for 18 h at 16 °C. After cell fragmentation, the samples were centrifuged at 4000 rpm for 10 min, and then the supernatant and precipitate were collected for SDS-PAGE analysis and xylanase activity measurement. The xylanase activity was measured via the 3,5-dinitrosalicylic acid (DNS) method with some modifications [58]. As the substrate of xylanase, xylan (Sigma, St. Louis, MO, USA, 9014-63-5) was dissolved in 0.1 mol/L sodium acetate buffer (pH 5.0) in the concentration of 2 mg/mL. A total 800 µL of such a substrate solution was mixed with 200 µL samples (the supernatant of the induced bacterial solution) and incubated at 50 °C for 10 min. The reaction was terminated by adding 1.5 mL DNS before being boiled in a boiling water bath for 5 min. Standard curves were set by using 0, 0.2, 0.4, 0.6, 0.8, and 1.0 mL of 2 mg/mL xylose in 0.1 M sodium acetate, which was diluted to 2 mL by using 0.1 M sodium acetate buffer. After adding 3 mL DNS, the samples were incubated in a boiling water bath for 10 min before being diluted to 15 mL with water. In addition, 0.1 mol/L sodium acetate buffer was used as reagent blanks. The OD value at OD 540 nm was measured by using a UV-8000S spectrophotometer (Shanghai Yuanxi Instrument Co., Ltd.). Furthermore, 1.0 U represents the amount of enzyme that is required for producing 1.0 µmol of xylose per 1 min. 

### 4.7. Determination of Water Transport and Leaf Water Loss

Stems were taken from six-week-old wild-type and *Zmhsl-1* plants with similar development phases and placed upright in a 5% red ink solution. Photos were taken every 10 min to observe and record water transport [19]. Hand-cut sections were prepared at the same position in each stem and photographed. The leaves of the 6-week-old wild-type and *Zmhsl-1* plants were taken at the same position from each plant, and their fresh weights were measured every 30 min for a duration of 6 h for the analysis of water loss. The experiment was conducted with three independent replicates. The formula for calculating the rate of water loss is water loss rate = (Ws − Wt)/Ws × 100 (Ws: fresh leaf weight; Wt: leaf weight at a specified time t).

### 4.8. Lignin Staining

At the jointing stage, the penultimate fifth leaf was taken, embedded in 3% agarose, and sliced by using a VT1000 S microtome (Leica, Wetzlar, Germany). To the sections on a glass slide, 5% phloroglucinol was added dropwise, together with an equal volume of concentrated HCl. After allowing for a reaction for 2–4 min, the image of the stained section was observed and photographed under a Leica MZ16F stereo microscope (Leica, Wetzlar, Germany) [59]. 

### 4.9. Determination of Cell Wall Composition

At the jointing stage, the penultimate fifth leaf was taken, and the cellulose content was determined by the sulfuric acid and potassium dichromate oxidation method, as previously described [60]. The reduced sugar content was determined by the DNS method and converted into hemifiber content, as previously described [61]. Lignin content was determined by using the Klason method [61].

### 4.10. RNA Sequencing (RNA-Seq) and Data Analysis

At the VT stage, the penultimate fifth leaf above the ear was sampled in the morning (24 °C) and at noon (34 °C) for RNA-seq. The transcriptome sequencing and analysis were conducted by OE Biotech Co., Ltd. (Shanghai, China). Total RNA was extracted using the mirVana miRNA Isolation Kit (Ambion, Waltham, MA, USA) following the manufacturer’s protocols. RNA integrity was evaluated using the Agilent 2100 Bioanalyzer (Agilent Technologies, Santa Clara, CA, USA). The samples with RNA Integrity Number (RIN) ≥7 were subjected to the subsequent analysis. The libraries were constructed using TruSeq Stranded mRNA LTSample Prep Kit (Illumina, San Diego, CA, USA) according to the manufacturer’s instructions. Then, these libraries were sequenced on the Illumina sequencing platform (HiSeqTM 2500 or Illumina HiSeq X Ten), and 125 bp/150 bp paired-end reads were generated. To ensure the reliability and accuracy of the results, we performed Benjamini Hochberg correction on all *p*-values to calculate the False Discovery Rate (FDR). This method helps to control the problem of multiple comparisons and reduce the occurrence of false positive results. In this study, we chose FDR < 0.05 as the significance threshold, so only genes that met this criterion were considered significantly differentially expressed genes. Raw data (raw reads) were processed using Trimmomatic [62]. The reads containing ploy-N and the low-quality reads were removed to obtain the clean reads. Then, the clean reads were mapped to the reference genome using hisat2 [63]. The raw RNA-seq data that support the findings of this study have been deposited into the CNGB Sequence Archive (CNSA) of China National GeneBank DataBase (CNGBdb) with accession number CNP0005867.

## 5. Conclusions

In this study, a new maize heat-sensitive mutant, *Zmhsl-1*, was identified and characterized, and its causative gene *ZmHSL* was unravelled. *ZmHSL* mutation leads to a decrease in lignin content and alterations in cell wall structure, hindering water transport and affecting plant growth and development. Transcriptomic analysis elucidates an eclectic array of key genes that are involved in the responsive process of maize to high-temperature stress, providing important clues for formulating molecular breeding and genetic modification strategies to improve high-temperature resistance in maize.

## Figures and Tables

**Table 1:** Results of χ^2^ test of segregation ratios in F2 and BC1F1 populations under high temperature.

Population	Normal Leaf	Curling Leaf	Ratio	χ^2^
Zheng58 × *Zmhsl-1* F_2_	174	51	3.4:1	0.53
(Zheng58 × *Zmhsl-1*) × *Zmhsl-1* BC_1_F_1_	134	142	0.9:1	0.18

**Table 2:** Candidate gene list.

Gene ID	Chr	Position	Nucleotide Change	Codon Change	Type
*Zm00001d013540*	5	13935295	G>A	TGC>TAC	Missense-variant, Cys401Tyr
*Zm00001d013561*	5	14348384	C>T	CAG>TAG	Stop-gained, Gln680*

## Data Availability

All data generated or analysed during this study are available within the article or upon request from the corresponding author.

## Supplementary Materials

The following supporting information can be downloaded at https://www.mdpi.com/article/10.3390/ijms25168834/s1.
