# Supplementary material for: Function Analysis of a Maize Endo-1,4-β-xylanase Gene ZmHSL in Response to High-Temperature Stress
Source: Int J Mol Sci. 2024 Aug 14;25(16):8834. doi: 10.3390/ijms25168834 (PMC11354693; doi:10.3390/ijms25168834)
Supplement: Supplementary file 1 [file ijms-25-08834-s001.zip › ijms-3039812-supplementary.pdf]

Supplementary Figures

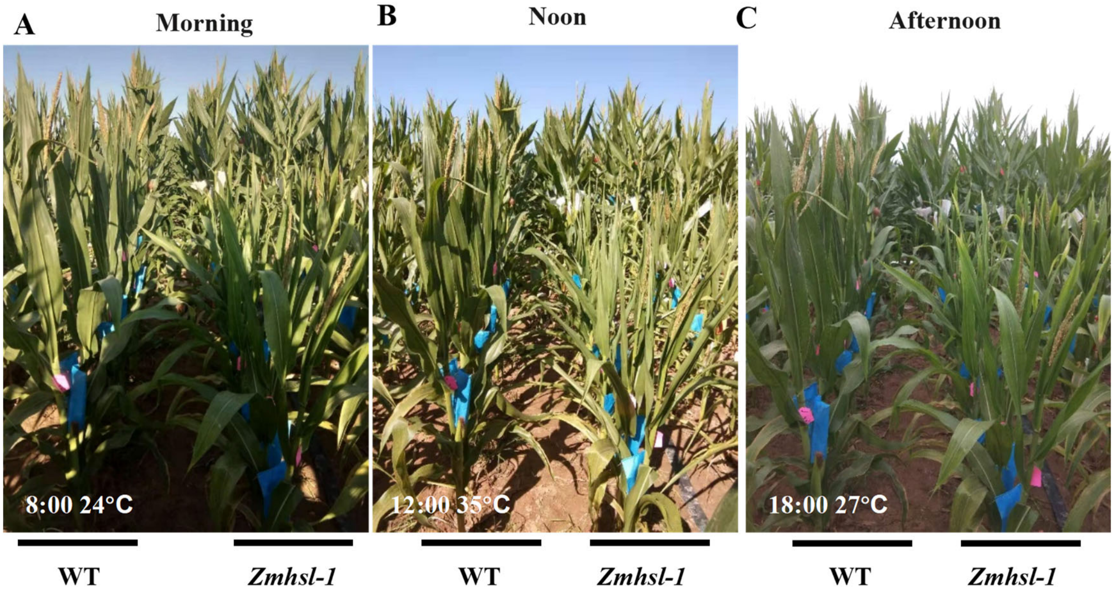

Figure S1. Comparison of wild type and *Zmhsl-1* during the day in summer at silking stage.

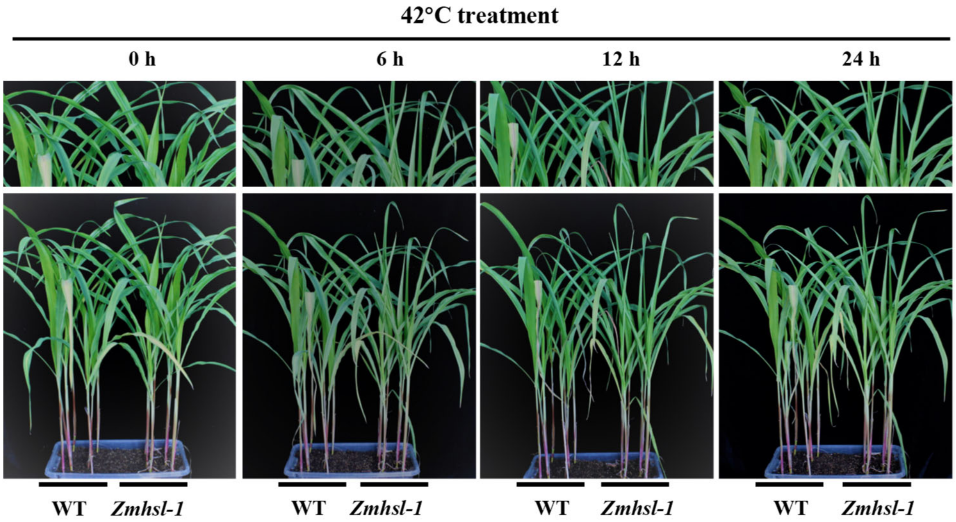

Figure S2. Comparison of wild-type and *Zmhsl-1* plants under heat stress in controlled growth chamber.

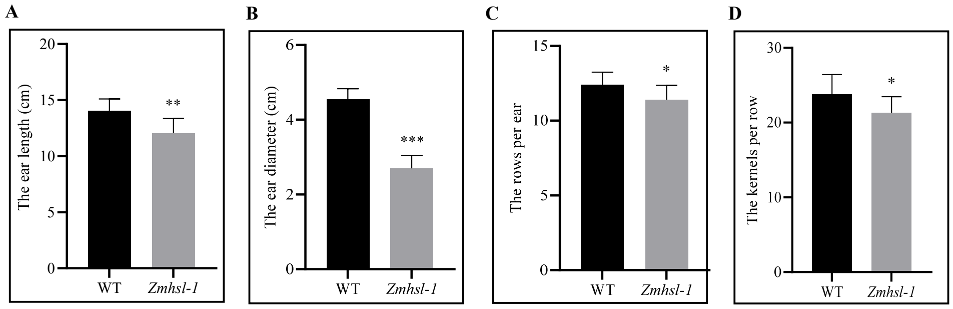

Figure S3. Phenotypic analysis of ear agronomic traits of wild type and *Zmhsl-1* plants. Comparison of wild-type (Z58) and *Zmhsl-1* plants on ear length **(A)**, ear diameter **(B)**, row number per ear **(C)** kernel number per row **(D)**. n = 10, n represents the number of samples; t-test (*P < 0.05, **P < 0.01, ***P < 0.001).

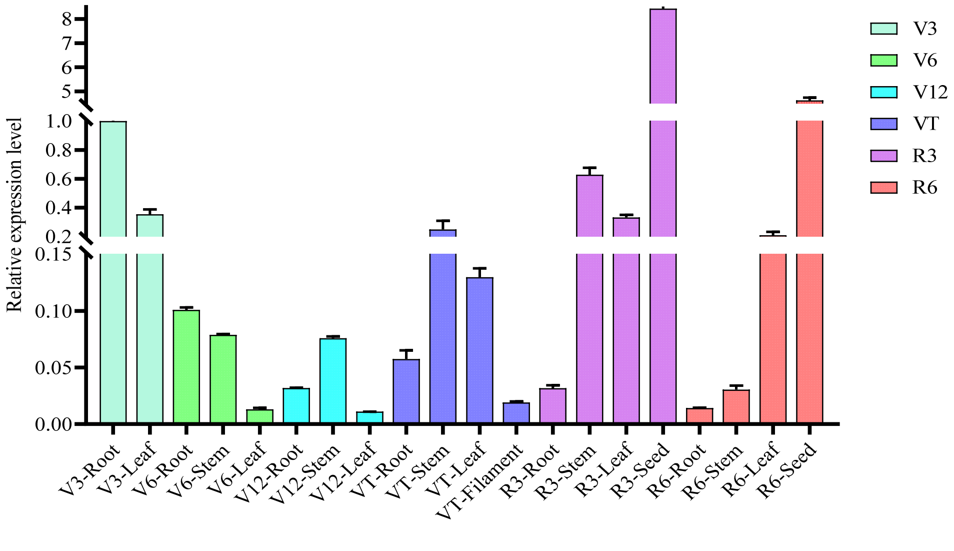

Figure S4. Spatiotemporal expression analysis of *ZmHSL*. Maize *tubulin* was used as an internal reference primer, and the expression level of *ZmHSL* in roots at V3 stage was set to 1.0.

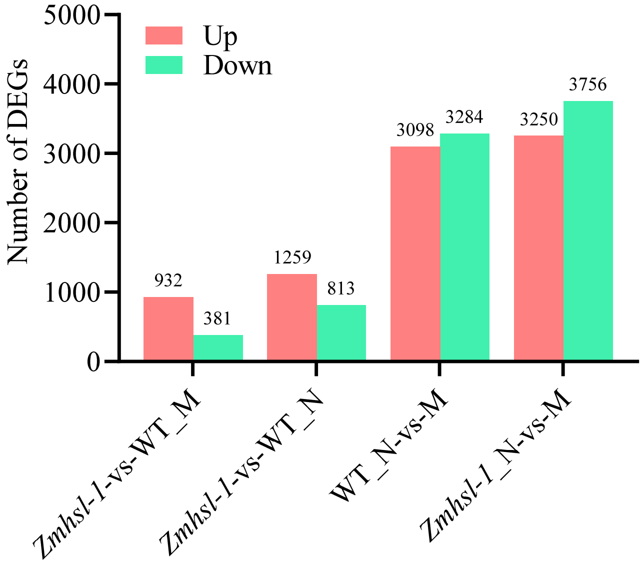

Figure S5. Statistics of differentially expressed genes.
